# Nitrogen‐Doped Graphene‐Like Carbon Intercalated MXene Heterostructure Electrodes for Enhanced Sodium‐ and Lithium‐Ion Storage

**DOI:** 10.1002/advs.202402708

**Published:** 2024-06-03

**Authors:** Kun Liang, Tao Wu, Sudhajit Misra, Chaochao Dun, Samantha Husmann, Kaitlyn Prenger, Jeffrey J. Urban, Volker Presser, Raymond R. Unocic, De‐en Jiang, Michael Naguib

**Affiliations:** ^1^ Department of Physics and Engineering Physics Tulane University New Orleans LA 70118 USA; ^2^ Department of Chemistry University of California Riverside CA 92521 USA; ^3^ Center for Nanophase Materials Sciences Oak Ridge National Laboratory Oak Ridge TN 37831 USA; ^4^ The Molecular Foundry Lawrence Berkeley National Laboratory Berkeley CA 94720 USA; ^5^ INM – Leibniz Institute for New Materials Campus D2 2 66123 Saarbrücken Germany; ^6^ Department of Materials Science and Engineering Saarland University Campus D2 2 66123 Saarbrücken Germany; ^7^ saarene – Saarland Center for Energy Materials and Sustainability Campus C4 2 66123 Saarbrücken Germany; ^8^ Department of Chemistry Tulane University New Orleans LA 70118 USA; ^9^ Present address: The State Key Laboratory of Fine Chemicals School of Chemical Engineering Dalian University of Technology Dalian 116024 P. R. China; ^10^ Present address: Department of Chemical and Biomolecular Engineering Vanderbilt University Nashville TN 37212 USA

**Keywords:** batteries, energy storage, graphene, heterostructures, MXene

## Abstract

MXene is investigated as an electrode material for different energy storage systems due to layered structures and metal‐like electrical conductivity. Experimental results show MXenes possess excellent cycling performance as anode materials, especially at large current densities. However, the reversible capacity is relatively low, which is a significant barrier to meeting the demands of industrial applications. This work synthesizes N‐doped graphene‐like carbon (NGC) intercalated Ti_3_C_2_T*_x_* (NGC‐Ti_3_C_2_T*_x_*) van der Waals heterostructure by an in situ method. The as‐prepared NGC‐Ti_3_C_2_T*_x_* van der Waals heterostructure is employed as sodium‐ion and lithium‐ion battery electrodes. For sodium‐ion batteries, a reversible specific capacity of 305 mAh g^−1^ is achieved at a specific current of 20 mA g^−1^, 2.3 times higher than that of Ti_3_C_2_T*_x_*. For lithium‐ion batteries, a reversible capacity of 400 mAh g^−1^ at a specific current of 20 mA g^−1^ is 1.5 times higher than that of Ti_3_C_2_T*_x_*. Both sodium‐ion and lithium‐ion batteries made from NGC‐Ti_3_C_2_T*_x_* shows high cycling stability. The theoretical calculations also verify the remarkable improvement in battery capacity within the NGC‐Ti_3_C_2_O_2_ system, attributed to the additional adsorption of working ions at the edge states of NGC. This work offers an innovative way to synthesize a new van der Waals heterostructure and provides a new route to improve the electrochemical performance significantly.

## Graphical abstract

Multilayer MXenes, noted for their metal‐like conductivity and layered structure, shows limited reversible capacity in energy storage. To overcome this, N‐doped graphene‐like carbon (NGC) is confined in between Ti3C2Tx layers to form a heterostructure. This structure significantly improves the capacity and stability of sodium‐ion and lithium‐ion batteries, offering a promising method to enhance electrochemical performance.

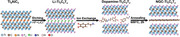

## 1 Introduction

Since transition metal carbides and/or nitrides, MXenes, were discovered in 2011,^[^ ^1^ ^]^ their number has increased rapidly, and more than 50 different compositions have been synthesized to date.^[^ ^1^, ^2^, ^3^, ^4^ ^]^ MXenes show outstanding physical, chemical, electronic, and optical properties, giving rise to their use in many applications from electrochemical energy storage^[^ ^5^, ^6^ ^]^ to catalysis,^[^ ^7^, ^8^ ^]^ electronics,^[^ ^9^, ^10^, ^11^ ^]^ sensing,^[^ ^12^, ^13^, ^14^ ^]^ and bio‐related applications. Owing to its layered structure and metal‐like electrical conductivity (≈24 000 S cm^−1^),^[^ ^15^ ^]^ MXenes have been investigated as electrode materials for many different electrochemical energy storage systems, such as supercapacitors,^[^ ^16^, ^17^ ^]^ lithium‐ion batteries (LIBs),^[^ ^18^, ^19^ ^]^ beyond LIBs (Na^+^, K^+^, Mg^2+^, Ca^2+^),^[^ ^20^, ^21^, ^22^, ^23^ ^]^ and alkali ion‐capacitors.^[^ ^24^, ^25^ ^]^ MXenes exhibit very high rate handling capabilities (charge and discharge at high current densities), which is crucial for fast‐charging battery systems.^[^ ^26^, ^27^ ^]^ However, the reversible capacity is relatively low especially for multilayer MXenes, which is a significant barrier to meet for industrial use. For example, as sodium‐ion battery (SIB) electrodes, Ti_3_C_2_T*_x_*, Ti_3_CNT*_x_*, and Ti_2_C_0.5_N_0.5_T*_x_* show specific capacities of 103, 157, and 182 mAh g^−1^, respectively, at a rate of 20 mA g^−1^ in 1 m NaPF_6_ electrolyte in ethylene carbonate (EC)/diethyl carbonate (DEC) with 1:1 (by volume).^[^ ^21^ ^]^

Three approaches are usually explored to improve the charge storage capacity of MXene electrodes: oxidation of MXenes, introduction of external metal ions, and making MXene composites. i) Oxidation of MXenes: Metal oxides, such as TiO_2_, Nb_2_O_5_, and mixed‐metal oxides (like titanium niobium oxide^[^ ^19^ ^]^), are promising anode candidates for rechargeable batteries owing to their high capacity and low cost. 2D Ti_3_C_2_T*_x_* can be oxidized in air to synthesize TiO_2_ nanocrystals enmeshed in thin sheets of disordered graphitic carbon structures, which can handle very high cycling rates as LIBs anode material.^[^ ^4^ ^]^ ii) Introducing external metal ions: Sn(IV)‐modified/decorated Ti_3_C_2_T*_x_* and V_2_CT*_x_* were reported to achieve high specific capacities of 635 and 1284 mAh g^−1^ at a rate of 100 mA g^−1^, respectively.^[^ ^28^, ^29^ ^]^ iii) MXene composites: Arnold et al presented a guideline for designing an advanced hybrid antimony MXene compound for application in high‐performance SIBs, with a high reversible capacity of 450 mAh g^−1^ at 0.1 A g^−1^ with a capacity retention of ≈96% after 100 cycles.^[^ ^30^ ^]^ All the methods mentioned above improved the capacity; however, the cycling stability of the as‐prepared electrode was decreased. Therefore, a balance between high capacity and excellent cycling stability should be a goal for future research.

Considering the intercalation/deintercalation mechanism of MXene‐based energy storage, theoretical studies show that two layers of Li‐ions can be absorbed on the MXene surfaces, which allows fast ion diffusion leading to high capacity.^[^ ^30^, ^31^ ^]^ Theoretical studies predict that two layers of Li‐ions can be adsorbed, but in experimental practice, the stoichiometry does not reach this level.^[^ ^20^ ^]^ We hypothesize that intercalating MXene with another layer of electrically conductive material will allow for two alkali ions layers between the MXene sheets instead of one layer of alkali ions. Recently, we proved that the interlayer spacings could be tuned by intercalating polymeric chains with different chain lengths.^[^ ^32^ ^]^ Therefore, MXene heterostructures might be a good option to achieve high capacity with excellent cycling stability. Very recently, an in situ carbon intercalated Nb_2_CT*_x_* was reported to achieve significantly improved electrochemical performance as a potassium‐ion battery electrode.^[^ ^33^ ^]^ Herein, we report an in situ method to synthesize an N‐doped graphene‐like carbon intercalated Ti_3_C_2_T*_x_* (NGC‐Ti_3_C_2_T*_x_*) heterostructure and evaluate the electrochemical performance as SIBs and LIBs. For SIBs, a reversible specific capacity of 305 mAh g^−1^ was achieved at a specific current of 20 mA g^−1^, more than twice that of pristine Ti_3_C_2_T*_x_* MXene. For LIBs, a reversible capacity of 400 mAh g^−1^ at a specific current of 20 mA g^−1^ is 1.5 times higher than that of pristine Ti_3_C_2_T*_x_* MXene. Both SIBs and LIBs show excellent cycling stability. In addition, theoretical calculations were performed to understand the capacity increase further. The maximum Na‐ion concentration in NGC‐Ti_3_C_2_O_2_ is 2.3 (maximum number of Na per unit cell), with a corresponding capacity of 289 mAh g^−1^, which is also close to the experimental result (≈305 mAh g^−1^) for Na‐ion intercalation. The extra Na‐ions could also adsorb on the edge sites around the NGC, accounting for a higher capacity than theoretical calculations predict. Similarly, the maximum Li‐ion concentration in NGC‐Ti_3_C_2_O_2_ is 3.0 (maximum number of Li per unit cell) with the corresponding capacity of 371 mAh g^−1^, which is also close to the experimental result (≈400 mAh g^−1^) for Li‐ion intercalation.

## 2 Results and Discussion

### 2.1 Materials Characterization

**Figure 1:**
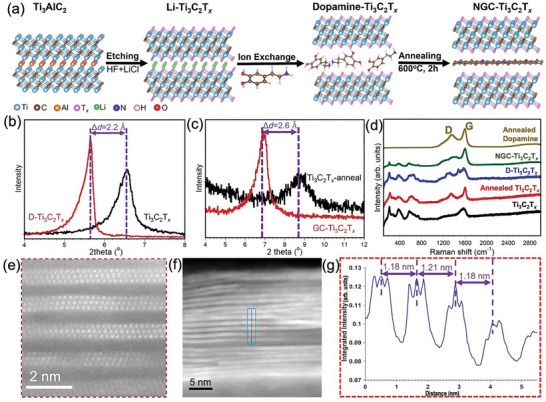
Synthesis process and material characterization of NGC‐Ti_3_C_2_T*_x_* samples. a) A schematic illustration of the preparation of NGC‐Ti_3_C_2_T*_x_*. X‐ray diffractograms of pristine Ti_3_C_2_T*_x_* and D‐Ti_3_C_2_T*_x_* b) before and c) after annealing at 600 °C for 2 h. d) Raman spectra of various samples. e,f) HAADF‐STEM images of NGC‐Ti_3_C_2_T*_x_* material and g) corresponding intensity line scan showing the interlayer spacing.

The process to prepare the NGC‐Ti_3_C_2_T*_x_* heterostructure material is schematically illustrated in **Figure** **1**a: i) A mixed solution of HF and LiCl was used to remove Al from Ti_3_AlC_2_ powders and intercalate Li^+^ between the layers of Ti_3_C_2_T*_x_* MXene, producing Li‐intercalated Ti_3_C_2_T*_x_* MXenes; ii) ion exchange from Li^+^ to protonated dopamine by immersing Li‐intercalated Ti_3_C_2_T*_x_* MXenes into dopamine hydrochloride solution, leading to dopamine cations intercalation in‐between the Ti_3_C_2_T*_x_* layers, yielding dopamine‐intercalated Ti_3_C_2_T_*x*_ (D‐Ti_3_C_2_T*_x_*) MXenes; iii) annealing the as‐prepared D‐Ti_3_C_2_T*_x_* MXenes at 600 °C for 2 h under Ar gas, to finally produce NGC‐Ti_3_C_2_T*_x_* MXenes. This temperature was chosen to ensure dopamine is decomposed to form NGC without causing recrystallizing Ti_3_C_2_T*_x_*.^[^ ^34^, ^35^, ^36^, ^37^ ^]^ As shown in Figure S1 (Supporting Information), the MAX phase peaks disappeared, and the MXenes peaks can be observed clearly, reflecting the successful removal of Al from Ti_3_AlC_2_ powders and conversion of the MAX phase into MXene. The successful intercalation of dopamine can be evaluated by monitoring the (002) peak position of MXene to determine the *d*‐spacing of D‐Ti_3_C_2_T*_x_* and NGC‐Ti_3_C_2_T*_x_* MXenes in comparison to pristine and annealed MXene. The *d*‐spacing can be calculated using Bragg's law, *d*  =  λ/2sinθ, where λ is the X‐ray wavelength (λ  =  0.1545 nm) and θ is the diffraction angle. As shown in Figure 1b, the *d*‐spacing shifts from 13.4 Å (6.6° 2θ) for pristine Ti_3_C_2_T*_x_* MXenes to 15.6 Å (5.7° 2θ) after the dopamine intercalation, which means the interlayer spacing expands by ≈2.2 Å, indicating that dopamine cations are intercalated in‐between the Ti_3_C_2_T*_x_* MXene layers. Compared to Ti_3_C_2_T*_x_* MXene after annealing, as displayed in Figure 1c, we can calculate that the *d*‐spacing of NGC‐Ti_3_C_2_T*_x_* MXene is 12.8 Å, which is ≈2.6 Å larger than annealed Ti_3_C_2_T*_x_*. The reduction in the *d*‐spacing upon annealing is not surprising, considering that in the temperature range of 100–600 °C, the removal of intercalated water and ‐OH termination groups bonding to Ti_3_C_2_T_x_ MXene leads to decreased *d*‐space.^[^ ^38^ ^]^

The Raman spectrum of Ti_3_C_2_T*_x_* MXene (bottom spectrum in Figure 1d) shows peaks at 210, 375, 580, and 740 cm^−1^, which are slightly different from Ti_3_C_2_T*_x_* MXene after HF treatment,^[^ ^3^, ^4^ ^]^ because of the different surface terminations.^[^ ^19^ ^]^ All Ti_3_C_2_T*_x_* MXene Raman peaks can be observed in annealed Ti_3_C_2_T*_x_*, D‐Ti_3_C_2_T_x_, and NGC‐ Ti_3_C_2_T*_x_* MXenes in Figure 1d. In D‐Ti_3_C_2_T*_x_*, dopamine peaks can be found at 653, 1326, and 1480 cm^−1^, consistent with the reported dopamine Raman data.^[^ ^39^, ^40^ ^]^ XRD and Raman data confirm that dopamine was successfully intercalated between the Ti_3_C_2_T*_x_* MXene sheets. Raman spectrum of as synthesized MXene (Figure 1d) shows the characteristic D‐band and G‐band of incompletely graphitic carbon. This may be related to carbon impurities in the MAX phases or from over‐etching MXene leading to the small amount of carbide‐derived carbon. The D and G peaks of annealed MXene sample showed a blueshift and decreased width, indicating an increased degree of graphitization. The reasons for this graphitization are currently not clear and warrant further investigations. Considering that graphitization of amorphous carbon typically requires much higher temperatures, this suggests that MXene might act as a catalyst to lower the graphitization temperature of carbon. This observation can be explained that during annealing, the surface terminations (OH, F, O groups) on the Ti_3_C_2_T*_x_* MXene sheets decompose and are removed. This leaves behind a carbon‐rich surface. The carbon atoms originally within the MXene structure become freer to rearrange without the steric hindrance of surface terminations. The thermal energy provided by annealing allows the carbon atoms to gradually reorder into more extended conjugated aromatic clusters and domains within the MXene sheets. Defects within the original carbon lattice are gradually eliminated through a recrystallization process driven by the annealing. This increased degree of graphitization leads to the blueshift and sharpening of the D‐band and G‐band peaks observed in the Raman spectra after annealing Ti_3_C_2_T*_x_* MXene. In addition, the D‐band and G‐band intensity ratio, I_D_/I_G_, of annealed dopamine, annealed Ti_3_C_2_T*_x_*, and NGC‐Ti_3_C_2_T*_x_* MXenes are estimated as 0.94, 0.80, and 0.88, respectively, indicating the existence of disordered graphitic carbon in annealed dopamine and NGC‐Ti_3_C_2_T*_x_* MXenes. Furthermore, the I_D_/I_G_ of 0.88 in NGC‐Ti_3_C_2_T*_x_* MXene aligns with the I_D_/I_G_ value of 0.9 reported for stacked MoS_2_ and N‐doped graphene.^[^ ^41^ ^]^ Considering the high I_D_/I_G_ ratio, possibly due to highly disordered carbon, but still the nanoconfined nature of the carbon between MXene layers, we have used “graphene‐like” to describe the intercalated carbon species. Exploring oxygen‐free carbon sources as intercalants might lead to a more ordered carbon confined between the layers.

The NGC‐Ti_3_C_2_T*_x_* MXene structure was studied using high‐angle annular dark‐field (HAADF) scanning transmission electron microscopy (STEM) imaging, as shown in Figure 1e,g. Low and high‐magnification STEM images in Figure 1e,f reveals the typical layered Ti_3_C_2_T*_x_* structure. The interlayer spacing between NGC‐Ti_3_C_2_T*_x_* MXene layers was measured to be ≈1.2 nm using line intensity profile measurements from the region marked in Figure 1e,f, which agrees with the XRD results (1.28 nm). However, we still can observe that the interlayer spacings were not the same for different locations due to the different morphologies of intercalated nanoflakes. During the annealing process, the intercalated dopamine cations acted as carbon and nitrogen sources and decomposed in their respective locations within the confined MXene interspace. The decomposed products (viz., C and N atoms) may segregate and precipitate out as nanoflakes due to their confinement and limited solubility in MXene. The MXene surface or defects/edges may catalyze the localized graphitization of intercalated C and N precursors, leading to nanoflaked morphology rather than a homogeneous intercalation compound. Additionally, differences in thermal expansion between MXene layers and intercalated species could induce internal stresses during annealing, driving the nanoflaked self‐assembly to relieve stress.

**Figure 2:**
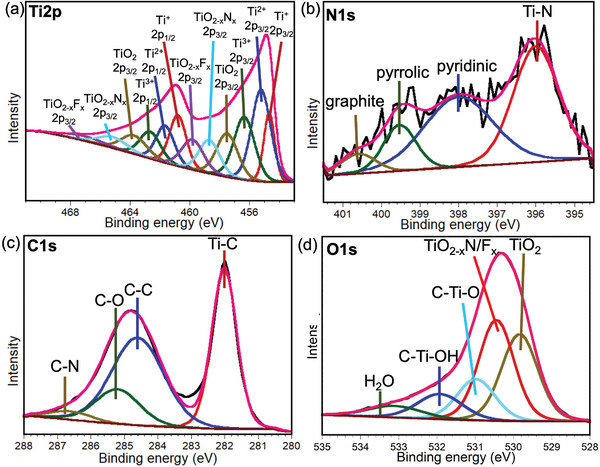
XPS analysis of NGC‐Ti_3_C_2_T*_x_*. a,d) High‐resolution XPS spectra of Ti 2p, N 1s, C1s, O1s in NGC‐Ti_3_C_2_T*_x_*.

X‐ray photoelectron spectroscopy (XPS) measurements were carried out to investigate chemical states of the elements of NGC‐Ti_3_C_2_T*_x_* MXene. From the XPS survey shown in Figure S2a (Supporting Information), Ti, C, and N were detected with surface terminations containing O and F. The weak Al peak demonstrates the removal of Al to form multilayer Ti_3_C_2_T*_x_* MXene. After analyzing the high‐resolution elements spectra, as displayed in **Figure** **2**a, Ti 2p peak can be deconvoluted to +1, +2, +3, and +4 oxidation states, corresponding to ‐O/‐OH/‐F terminations. 11% photoemission of Ti‐ON is noted in the Ti 2p peak, indicating N can terminate the MXene surfaces when dopamine is decomposed during the annealing treatment. High‐resolution N 1s region spectra show four fitted peaks at 396.4, 398.4, 399.9, and 401.0 eV, which correspond to Ti‐ON and pyridinic/pyrrolic/graphitic N from N‐doped carbon species, respectively. 43% photoemission of Ti‐ON is found in the N 1s peak (Table S1, Supporting Information), confirming the existence of ‐ON termination. The fractions of pyridinic/pyrrolic/graphitic N are 40%, 12%, and 6%, respectively. Large amounts of pyridinic and pyrrolic N fractions indicate most of the N are located at the edge or surface hole defect sites in a graphene‐like carbon layer.^[^ ^42^ ^]^ As presented in Figure 2c, the C 1s spectrum can be fitted by four peaks, including a C─N peak at 286.4 eV with 3% photoemission, demonstrating the existence of N‐doped carbon species. All of the above supports that we have synthesized the NGC‐Ti_3_C_2_T*_x_* MXenes. For the O1s region in Figure 2d, we can observe the Ti‐ON or Ti‐OF terminations. As presented in Figure S2b (Supporting Information) shows both Ti─F and Ti‐OF terminations. More XPS details, including peak positions, full width at half maximum, FWHM, and fractions, are summarized in Table S1 (Supporting Information).

### 2.2 Sodium‐Ion Battery Application

**Figure 3:**
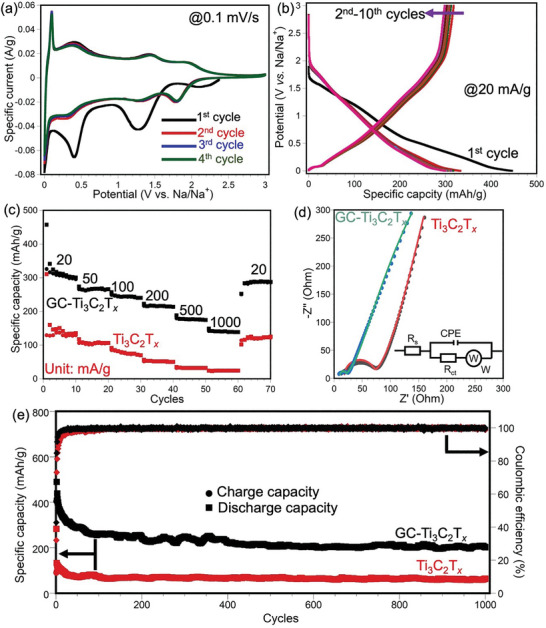
Electrochemical performance of NGC‐Ti_3_C_2_T*_x_* as a Na‐ion battery electrode material. a) Cyclic voltammograms of NGC‐Ti_3_C_2_T*_x_* for the first four cycles at a scan rate of 0.1 mV s^−1^. b) Galvanostatic charge/discharge testing of NGC‐Ti_3_C_2_T*_x_* for the first ten cycles at a specific current of 20 mA g^−1^. c) Rate capabilities of NGC‐Ti_3_C_2_T*_x_* and Ti_3_C_2_T*_x_* as Na‐ion battery electrodes. d) Nyquist plots NGC‐Ti_3_C_2_T*_x_* and Ti_3_C_2_T*_x_*. Inset is the equivalent circuit. The dots are the original data, and the solid line are the fitting curves. e) Long‐term cycling performance at a current density of 200 mA g^−1^.

To investigate the electrochemical energy storage performance of as‐prepared NGC‐Ti_3_C_2_T*_x_* MXene, standard CR2032 SIBs configurations were assembled with NGC‐Ti_3_C_2_T*_x_* MXene as working electrode and Na foil as counter/reference electrodes in 1 m NaPF_6_/EC‐DEC electrolyte. **Figure** **3**a shows the cyclic voltammetry (CV) curves of NGC‐Ti_3_C_2_T*_x_* MXene during the first four cycles at a scan rate of 0.1 mV s^−1^ between 0.01 and 3.0 V versus Na/Na^+^. Three broad peaks located at ≈0.4, 1.25, and 2.1 V are exhibited in the first cathodic scan, but they disappear in the following cycles; therefore, they can be ascribed to the formation of the solid electrolyte interphase (SEI), and/or irreversible reactions with surface terminations. The cyclic voltammograms almost overlap from the second to the fourth cycle, indicating a highly reversible electrochemical behavior. For the first cycle, the cyclic voltammogram shape of NGC‐Ti_3_C_2_T*_x_* (Figure 3a) MXene is different from the one of Ti_3_C_2_T*_x_* MXene Figure S3, Supporting Information), suggesting NGC plays an essential role in the electrochemical performance of NGC‐Ti_3_C_2_T*_x_* MXene. Compared to the cyclic voltammogram of Ti_3_C_2_T*_x_* MXene, the peaks at 0.4 V and 2.1 V are observed in both materials, while the peak at ≈1.25 V is pronounced in NGC‐Ti_3_C_2_T*_x_* only; this can be assigned to either ‐ON terminated MXene or the NGC, indicated by its undeniable portion in the N 1s spectra. For cycles beyond the first, three pairs of redox peaks are noted at 0.31/0.40, 1.28/1.43, and 1.80/1.95 V versus Na/Na^+^ for NGC‐Ti_3_C_2_T*_x_* MXene, while there are no pronounced redox peaks for Ti_3_C_2_T*_x_* MXene.

After NGC intercalation, the interlayer spacing of NGC‐Ti_3_C_2_O_2_ increases by ≈0.6 Å compared with Ti_3_C_2_O_2_. This provides additional space to store Na‐ions, thereby playing a crucial vital in enhancing the capacity of Na‐ion battery. Additionally, the existence of ‐ON termination has been confirmed in the N 1s peak, which contributes to the formation of an additional three redox sites of reactions. This is further demonstrated by the appearance of three pairs of redox peaks at 0.31/0.40, 1.28/1.43, and 1.80/1.95 V versus Na/Na^+^ for NGC‐Ti_3_C_2_T*_x_* MXene, while no pronounced redox peaks are observed for Ti_3_C_2_T*_x_* MXene. Furthermore, as reported, N‐groups exhibit the sodiophilicity, and thus absorbing more Na‐ions,^[^ ^43^ ^]^ which corresponds with the calculated results, that is, a maximum of 2.3 Na per NGC‐Ti_3_C_2_O_2_ unit cell.

Galvanostatic charge/discharge (GCD) voltage profiles of NGC‐Ti_3_C_2_T*_x_* MXene at a specific current of 20 mA g^−1^ are shown in Figure 3b. The initial discharge process delivers a specific capacity of 447 mAh g^−1^, which then decreases to 317 mAh g^−1^ for the first charge process, showing a Coulombic efficiency of 71%. The specific capacity fading in the first sodiation and de‐sodiation steps is attributed to SEI formation and other irreversible reactions between sodium, the NGC, and/or the MXene surface terminations. A reversible specific capacity of 305 mAh g^−1^ is achieved after five cycles.

GCD testing was conducted at different specific currents to further evaluate the electrochemical performance of NGC‐Ti_3_C_2_T*_x_* MXene as electrodes for SIBs. As illustrated in Figure 3c, the NGC‐Ti_3_C_2_T*_x_* MXene electrode shows reversible specific capacities of 305, 266, 243, 216, 176, and 140 mAh g^−1^ at specific currents of 20, 50, 100, 200, 500, and 1000 mA g^−1^, respectively. The specific capacity increased to 286 mAh g^−1^ when the specific current returned to 20 mA g^−1^, suggesting a high‐rate handling capability and sodiation/de‐sodiation reversibility. Compared to Ti_3_C_2_T*_x_* MXene, the specific capacities of NGC‐Ti_3_C_2_T*_x_* MXene increased 2.3‐6.0 times when the specific currents increase from 20 to 1000 mA g^−1^, reflecting intercalating NGC is a promising approach to increase the specific capacity, especially at high specific currents. It can be ascribed to N atoms on the intercalated NGC layers providing more Na ions absorption sites, amalgamated with expanded d‐spacing for expedited ions transport.

For most electrode materials, the total charge storage encompasses a balance of surface and diffusion contributions, which varies with operating conditions and material properties. Understanding both aspects is crucial for characterizing charge storage performance. Surface‐controlled processes pertain to charge storage mechanisms that occur at or near the electrode/electrolyte interface, such as double layer capacitance, pseudocapacitance, and surface redox reactions. Diffusion‐controlled processes refer to charge storage mechanisms that require ion insertion/extraction into the bulk electrode material, such as intercalation reactions in batteries. The response from the peak current in curves and the scan rate can be described by a power law as follows:^[^ ^32^, ^44^ ^]^

(1) $$i_{p}=av^{b}$$

where *i*_p_ is the peak current, *v* is the scan rate, *a* and *b* are adjustable parameters. Here, we can use the *b* value to reflect the rate‐determining step. Specifically, *b* = 1.0 indicates a surface‐controlled step, while *b* = 0.5 donates a semi‐infinite diffusion‐controlled step. As illustrated in Figure S4 (Supporting Information), the *b* values were calculated to be ≈0.62 for NGC‐Ti_3_C_2_T*_x_* MXene, corresponding to a combined control step between surface‐controlled processes and diffusion‐controlled processes. In contrast, the *b* values for Ti_3_C_2_T*_x_* MXene were calculated to be ≈0.5, suggesting a mainly diffusion‐controlled system. Determining the exact mechanism would require analysis of the material/system properties and could be complex. However, diffusion still plays a significant role in the rate determination.

To investigate the kinetics of NGC‐Ti_3_C_2_T*_x_* MXene, electrochemical impedance spectroscopy (EIS) was conducted in the frequency range from 100 to 100 kHz. As displayed in Figure 3d, a semicircle in the high‐to‐medium‐frequency range and a straight line in the low‐frequency range can be observed in Nyquist plots of Ti_3_C_2_T*_x_* and NGC‐Ti_3_C_2_T*_x_* MXene electrodes. To further understand the EIS, the inset in Figure 3d shows an equivalent circuit, including the system resistance (R_s_), charge transfer resistance (R_ct_), Warburg resistance (W), and a constant phase element (CPE). The NGC‐Ti_3_C_2_T*_x_* MXene electrode shows smaller estimated system resistance (6.5 Ω) and charge transfer resistance (16.9 Ω) than those for the Ti_3_C_2_T*_x_* MXene electrode (of 11.1–62.4 Ω, respectively), demonstrating that intercalating NGC can facilitate the charge transfer and ion diffusion to enhance the electrochemical performance.

As shown in Figure 3e, the as‐prepared Ti_3_C_2_T*_x_* and NGC‐Ti_3_C_2_T*_x_* MXenes present stable specific capacities of 85 and 256 mAh g^−1^, respectively, after 90 cycles at a specific current of 200 mA g^−1^, suggesting excellent cycling performance after intercalating NGC nanoflakes. The NGC‐Ti_3_C_2_T*_x_* MXene electrode shows higher Coulombic efficiency than Ti_3_C_2_T*_x_* MXene in the initial sodiation/de‐sodiation stage, indicating highly efficient electrochemical redox processes after intercalating NGC.

### 2.3 Lithium‐Ion Battery Application

We also tested NGC‐Ti_3_C_2_T*_x_* MXene as an electrode for LIB application. Similar to the results from SIB testing, the electrochemical performance of LIB is improved by intercalating NGC. As shown in Figure S5a (Supporting Information), NGC‐Ti_3_C_2_T*_x_* MXene electrode shows an initial discharge capacity of 504 mAh g^−1^, and achieves a reversible capacity of 400 mAh g^−1^ after five cycles at a specific current of 20 mA g^−1^. The NGC‐Ti_3_C_2_T*_x_* MXene electrode shows reversible specific capacities of 400, 350, 325, 304, 267, and 235 mAh g^−1^ at specific currents of 20, 50, 100, 200, 500, and 1000 mA g^−1^. The specific capacity came back to 392 mAh g^−1^ when the specific current returned to 20 mA g^−1^, demonstrating the high‐rate handling capability and lithiation/de‐lithiation reversibility. Compared to Ti_3_C_2_T*_x_* MXene, the specific capacities of NGC‐Ti_3_C_2_T*_x_* Mxene increase ≈1.5 times when the specific currents increase from 20 to 1000 mA g^−1^. As shown in Figure S5c (Supporting Information), the as‐prepared NGC‐Ti_3_C_2_T*_x_* Mxene presents a stable specific capacity after 20 cycles at a specific current of 200 mA g^−1^, suggesting excellent cycling performance.

### 2.4 Li‐ and Na‐Ion Intercalation Modeling

Density functional theory (DFT) calculations were performed to understand the observed enhanced capacity. First, we constructed the structure of Ti_3_C_2_O_2_ as shown in Figure S6a (Supporting Information), and the calculated *c*‐lattice constant is 9.67 Å. After that, various ion intercalation configurations of Ti_3_C_2_O_2_ were optimized. We find that both the Li‐ and Na‐ions could intercalate into the interlayer of Ti_3_C_2_O_2_. The calculated maximum Li‐concentration “x” in the stoichiometric formula Li_x_Ti_3_C_2_O_2_ could reach 2, much greater than the maximum Na‐ion concentration of 1.125 in Na_x_Ti_3_C_2_O_2_. The corresponding configurations with different Li‐ or Na‐ion concentrations are shown in Figures S7 and S8 (Supporting Information), respectively. The capacities are estimated as 259 and 146 mAh g^−1^ for LIBs and NIBs with Ti_3_C_2_O_2_ electrodes, respectively. For comparison, the experimental capacities of Ti_3_C_2_O_2_ are 200 and 130 mAh g^−1^ for LIB and NIB, respectively.

**Figure 4:**
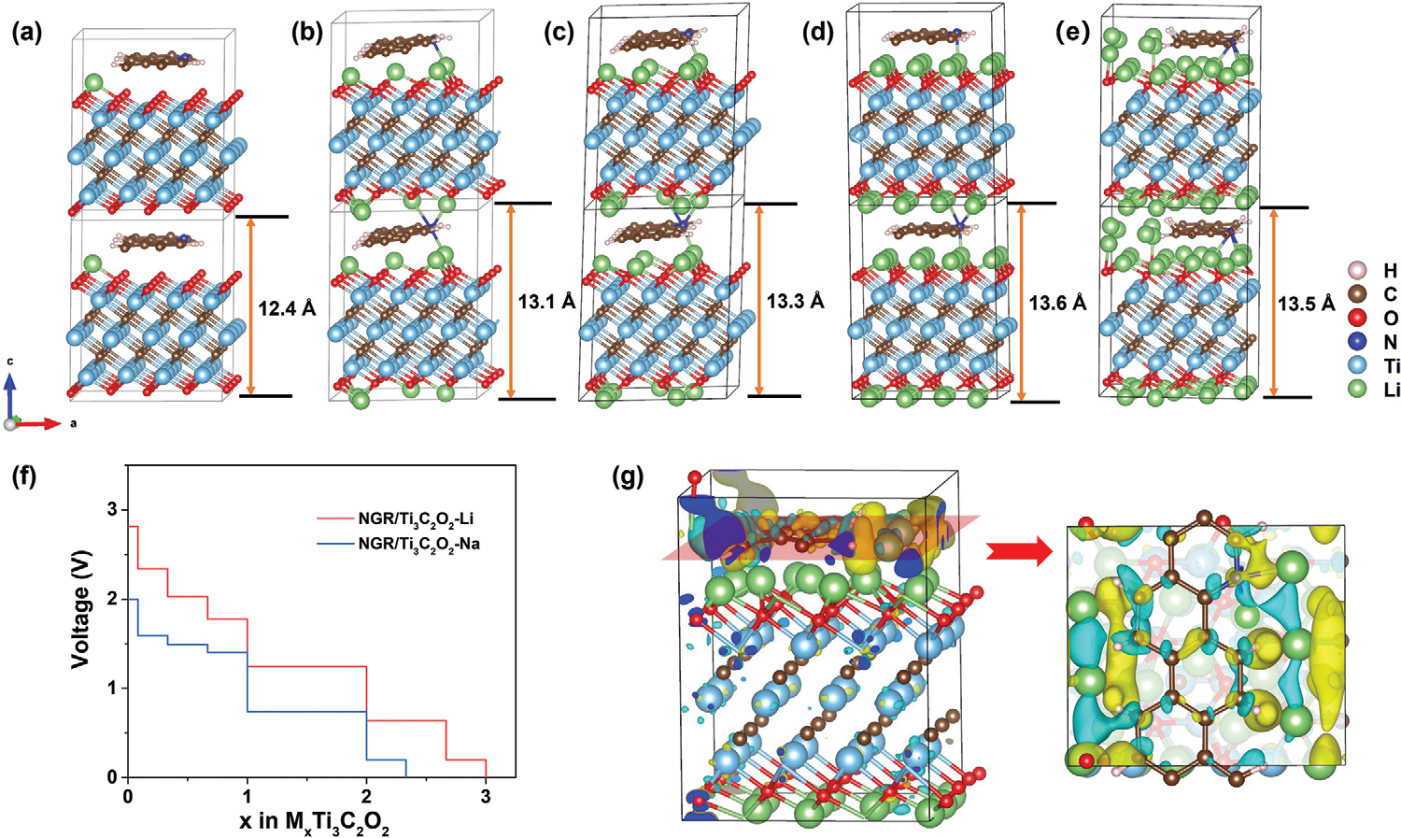
The optimized structures of NGC‐Ti_3_C_2_O_2_ with different Li‐ion concentrations: a) NGC‐Ti_3_C_2_O_2_‐0.083Li, b) NGC‐Ti_3_C_2_O_2_‐0.667Li, c) NGC‐Ti_3_C_2_O_2_‐1.0Li, d) NGC‐Ti_3_C_2_O_2_‐2.0Li, and e) NGC‐Ti_3_C_2_O_2_‐3.0Li, respectively. f) The calculated average voltage for Li‐ and Na‐ion intercalation into NGC‐Ti_3_C_2_O_2_. g) The electron‐density‐difference isosurfaces for Li‐ion intercalation around the edge of NGC (left, side view; right, top view). Isosurface value at 0.002 e Bohr^−^3: electron accumulation, yellow; electron depletion, cyan.

Our calculated results are consistent well with experimental results. The much higher capacity of Ti_3_C_2_O_2_ for Li‐ion intercalation aligns with the smaller ionic radius of Li‐ions than Na‐ions. In addition, as a benefit of the NGC intercalation in the structure, the interlayer spacing of NGC‐Ti_3_C_2_O_2_ increases to 12.3 Å compared to Ti_3_C_2_O_2_, as shown in Figure S6b (Supporting Information). The interlayer spacing increases by ≈0.6 Å with NGC in the interlayer, which is consistent well with the experimental result (10.2–12.8 Å) after dopamine intercalation. This interlayer spacing increase gives NGC‐T_3_C_2_O_2_ more space to store the Li‐ or Na‐ions. As shown in **Figure** **4**a‐d, the Li‐ion prefers to adsorb on the layers of Ti_3_C_2_O_2_ with a Li‐ion concentration of up to 2.0. After the complete adsorption of Li‐ions on the Ti_3_C_2_O_2_ layers, one extra Li‐ion could adsorb on the edge site around the NGC. The extra Li adsorption makes the maximum Li‐ion concentration x in NGC‐Li_x_Ti_3_C_2_O_2_ reach 3.0, as shown in Figure 4e, and the corresponding capacity is 371 mAh g^−1^. This estimated capacity value is close to the experimental result of ≈400 mAh g^−1^ for dopamine‐intercalated MXene.

Similarly, extra Na‐ions could also adsorb on the edge site around the NGC, as shown in Figure S9 (Supporting Information). The maximum Na‐ion concentration (maximum number of Na per unit cell) in NGC‐Na_x_Ti_3_C_2_O_2_ is 2.33, with the corresponding capacity of 289 mAh g^−1^, which is also close to the experimental result (≈300 mAh g^−1^) for Na‐ion intercalation. Based on various ion intercalation configurations in NGC‐Ti_3_C_2_O_2_, the calculated voltage profile is shown in Figure 4f for both Li‐ion and Na‐ion. The higher average voltage for Li‐ion intercalation in NGC‐Ti_3_C_2_O_2_ results in a higher energy density. To gain deeper insights, we plotted electron‐density‐difference isosurfaces during Li‐ion intercalation, as depicted in Figure 4g. This visualization clearly illustrates the transfer of electrons from Li (in the cyan region) to the NGC (in the yellow region) upon introducing extra Li ions. The accumulation of electrons along the periphery of the NGC suggests that the NGC can store some of these electrons. This property enhances the performance of NGC‐Ti_3_C_2_O_2_ significantly when compared to Ti_3_C_2_O_2_ alone.

## 3 Conclusion

In summary, novel NGC‐Ti_3_C_2_T*_x_* heterostructures were synthesized using an in situ method by carbonizing dopamine‐intercalated MXene. XRD, Raman, XPS, and STEM data confirm that NGC was intercalated between the MX‐layers of Ti_3_C_2_T*_x_*. When tested as electrodes for SIBs and LIBs, the as‐prepared NGC‐Ti_3_C_2_T*_x_* heterostructure showed a significantly improved electrochemical performance. For SIBs, a reversible specific capacity of 305 mAh g^−1^ is achieved at a specific current of 20 mA g^−1^, 2.3 times higher than that of Ti_3_C_2_T*_x_* MXene. For LIBs, a reversible capacity of 400 mAh g^−1^ at a specific current of 20 mA g^−1^ is 1.5 times higher than that of Ti_3_C_2_T*_x_* MXene. Theoretical calculations show that NGC‐Ti_3_C_2_T*_x_* heterostructure has more space for the Li‐ or Na‐ion storage, and the extra Li‐ or Na‐ions could adsorb on the edge site around the NGC. This work offers an innovative way to synthesize new heterostructures and provides a new route to improve electrochemical performance significantly.

## 4 Experimental Section

### Synthesis of NGC‐Ti_3_C_2_T_x_

Ti_3_AlC_2_ powders were prepared using a procedure described in the earlier work.^[^ ^32^ ^]^ In short, Ti (99.99%,−325 mesh, Alfa Aesar), aluminum (99.5%,−325 mesh, Alfa Aesar), and graphite (99.9995%,−325 mesh, Alfa Aesar) were mixed with a molar ratio of 3.00:1.20:1.88, then transferred into an alumina crucible and heated to 1600 °C at 10 °C min^−1^ for 2 h in an alumina tube furnace under a continuous Ar flow. The samples of D‐Ti_3_C_2_T_*x*_ were prepared through wet chemical etching and ion exchange. Briefly, 2 g of Ti_3_AlC_2_ powders (−325 mesh; <44 µm) were slowly added into 100 mL of 10 mass% aqueous hydrofluoric acid solution (HF, 48–51%, Acros Organics) and 2 g lithium chloride (LiCl, 99%, Fisher Scientific) in a 250 mL high‐density polyethylene bottle. The reaction temperature was maintained at 25 °C in an oil bath for 24 h. After that, the mixture was centrifuged at 3500 rpm to separate the powders and solution, and then the settled powders were washed using 6 m aqueous hydrochloride acid (HCl, 36.5–38%, Fisher Chemicals) and degassed deionized (DI) water for three times, respectively. The remaining sediments were transferred into 50 mL of 0.5 m dopamine hydrochloride (99%, Thermo Scientific) solution and rested for 4 days with daily manual shaking. Finally, the intercalated MXene was washed four times with DI water, and the materials were collected and dried using vacuum‐assisted filtration. The dried materials, referred to as D‐Ti_3_C_2_T*_x_*, were transferred into a tube furnace and annealed at 600 °C for 2 h with a heating rate of 5 °C min^−1^ under a continuous flow of argon, Ar, gas at a flow rate of 100 sccm. The resulting black powders were collected and labeled as NGC‐Ti_3_C_2_T*_x_*. For comparison, non‐intercalated Ti_3_C_2_T*_x_* after HF/LiCl etching was annealed under the same conditions.

### Materials Characterization

X‐ray diffraction was performed using a Rigaku DMAX2200 powder diffractometer with Cu‐Kα radiation (λ = 0.154 nm) at 40 kV and 40 mA. The step size and scan rate were set at 0.02° 2θ and 1°min^−1^, respectively.

XPS (Thermo Fisher) configured with an Al‐K_α_ excitation source was used to check the chemical states of NGC‐Ti_3_C_2_T*_x_*. Before collecting the XPS spectrum, the surfaces of the samples were cleaned by Ar^+^ sputtering for 5 min. The data were analyzed using the CasaXPS software.

Raman analysis was carried out using a Renishaw InVia microscope with a He‐Ne laser operating at 632.8 nm excitation wavelength, having a power of ≈0.1 mW at the focal point of the sample. For each sample, spectra from ten points were recorded with 20 s exposure time and accumulated five times. The microscope employed a 50x magnifying lens with a numeric aperture of 0.75. The samples were placed on a glass slide, and the system was calibrated with a silicon single crystal.

STEM imaging was performed using the Nion UltraSTEM100, equipped with a cold‐field emission gun and an aberration corrector on the probe‐forming lens. HAADF STEM images were acquired at an accelerating voltage of 60 kV, a convergence angle of 31 mrad, and a collection angle between 86–200 mrad. Before imaging, the specimens were baked at 160 °C for 8 h under vacuum to reduce surface contamination.

### Electrochemical Measurements

Standard two‐electrode CR2032 coin cells were used to evaluate the electrochemical performance of NGC‐Ti_3_C_2_T*_x_* material for SIBs and LIBs, in which NGC‐Ti_3_C_2_T*_x_* material was employed as working electrode and Na or Li foils as counter and reference electrodes for SIBs or LIBs, respectively. To prepare a slurry of NGC‐Ti_3_C_2_T*_x_* for casting of the working electrodes, as‐prepared NGC‐Ti_3_C_2_T*_x_* powder, carbon black (Super P, Imerys), and poly(vinylidene fluoride) (PVDF, average M_w_ ≈534 000 by GPC, Sigma–Aldrich) dissolved in N‐methyl‐2‐pyrrolidinone (NMP, 99.5%, Acros Organics, Extra Dry over Molecular Sieve) solvent were mixed thoroughly. The mass ratio of NGC‐Ti_3_C_2_T*_x_*:carbon black:PVDF was 8:1:1. The NGC‐Ti_3_C_2_T*_x_* working electrodes were prepared by drop‐casting the slurry on the carbon‐coated Cu foils, then dried at 60 °C in the oven overnight. The electrode mass loading was controlled at 1.2–1.8 mg cm^−2^, and we used glass fiber (GF/D, Whatman) separators. 1 m NaPF_6_ in EC/DEC with 1:1 ratio (by volume) and 1 m LiPF_6_ in EC/ethyl methyl carbonate (EMC) with 3:7 (by mass) were used as electrolytes for SIBs and LIBs, respectively. The assembling process was conducted in an Ar‐filled glovebox with O_2_ and H_2_O levels below 0.1 ppm. CV and GCD were conducted using a BioLogic VMP3 electrochemical workstation and Landt CT2001A cycler, respectively. The cut‐off electrochemical voltage windows of 0.001–3.0 V versus Na/Na^+^ or Li/Li^+^ were used for SIB and LIB, respectively. Electrochemical impedance spectroscopy with frequency from 100 kHz–10 mHz was conducted in a BioLogic VMP3 electrochemical workstation.

### Computational Methods

DFT calculations were performed using the Vienna Ab initio Simulation Package (VASP).^[^ ^45^, ^46^ ^]^ Interaction between valence electrons and the core was described by the projector augmented wave (PAW) method.^[^ ^47^, ^48^ ^]^ The electronic wave functions were expanded by the plane‐wave basis sets. The Perdew–Burke–Ernzerhof (PBE) functional of generalized gradient approximation was used for electron exchange‐correlation.^[^ ^46^ ^]^ The kinetic energy cut‐off was set to 500 eV for the plane waves. The Brillouin zone was sampled using Γ‐centered 6×10× 6 and 2×2×2 k‐point meshes for the Ti_3_C_2_O_2_ supercell and NGC/Ti_3_C_2_O_2_ heterostructure, respectively. To account for van der Waals interactions between MXene layers, we used Grimme's DFT + D3 method.^[^ ^49^ ^]^ The lattice parameters and atomic positions were fully relaxed until energy and force converged at 10^−5^ eV and 0.01 eV Å^−1^, respectively.

The supercell of Ti_3_C_2_O_2_ with an orthogonal lattice was constructed first and its optimized lattice constants are *a* = 10.42 Å; *b* = 6.02 Å, and *c* = 9.67 Å. Then, the heterostructure of NGC/Ti_3_C_2_O_2_ was constructed based on the structure of Ti_3_C_2_O_2_. In the constructed NGC/Ti_3_C_2_O_2_ structure, the NGC layer contains 15 C atoms and 1 N atom, while the Ti_3_C_2_O_2_ layer contains 36 Ti atoms. The ratio of C in NGC to Ti is ≈0.42, which matches the experimental result of 0.36. The optimized lattice constants of NGC/Ti_3_C_2_O_2_ are *a* = 10.40 Å, *b* = 8.92 Å, and *c* = 12.34 Å.

Based on the total energies of the NGC/Ti_3_C_2_O_2_ and its various intercalation configurations, the voltage profile for NGC/Ti_3_C_2_O_2_ electrode at 0 K with Li^+^ or Na^+^ intercalation was determined by the following equation:

(2) $$\begin{array}{cc} & V=\\ & -\frac{E\left(\mathrm{NGC}/M_{x_{2}}Ti_{3}C_{2}O_{2}\right)-E\left(\mathrm{NGC}/M_{x_{1}}Ti_{3}C_{2}O_{2}\right)-\left(x_{2}-x_{1}\right)E\left(M\right)}{\left(x_{2}-x_{1}\right)e},\;x_{2}>x_{1} \end{array}$$

where V is the calculated average voltage for Li^+^ or Na^+^ intercalation; E(NGC/M_x2_Ti_3_C_2_O_2_) and E(NGC/M_x1_Ti_3_C_2_O_2_) are the total energy of the NGC/M_x_Ti_3_C_2_O_2_ electrode with an alkali metal ion amount of x_2_ and x_1_, respectively; E(M) is the free energy per atom of a bulk alkali metal (Li: $R\bar{3}m$ and Na: $I\bar{4}3m$).

## Conflict of Interest

The authors declare no conflict of interest.

## Supporting information

Supporting Information

## Data Availability

The data that support the findings of this study are available from the corresponding author upon reasonable request.
