# Supplementary material for: Nitrogen‐Doped Graphene‐Like Carbon Intercalated MXene Heterostructure Electrodes for Enhanced Sodium‐ and Lithium‐Ion Storage
Source: Adv Sci (Weinh). 2024 Jun 3;11(31):2402708. doi: 10.1002/advs.202402708 (PMC11336969; doi:10.1002/advs.202402708)
Supplement: Supplementary file 1 — Supporting Information [file ADVS-11-2402708-s001.docx]

Supporting Information for:

**Nitrogen-doped, graphene-like carbon intercalated MXene heterostructure electrodes for enhanced sodium- and lithium-ion storage**

Kun Liang, Tao Wu^a^, SudhajitMisra, Chaochao Dun, Samantha Husmann, Kaitlyn Prenger,
Jeffrey J. Urban, Volker Presser, Raymond R. Unocic, De-enJiang^b^, Michael Naguib^*^

**Dr. Kun Liang, Dr. Kaitlyn Prenger, Prof. Michael Naguib**

Department of Physics and Engineering Physics, Tulane University, New Orleans, Louisiana 70118, United States of America.

* Corresponding’sauthorE-mail:[naguib@tulane.edu](mailto:naguib@tulane.edu)

**Dr. Tao Wu, Prof. De-en Jiang**

Department of Chemistry, University of California, Riverside, CA 92521, United States of America.

**Dr. SudhajitMisra, Dr. Raymond R. Unocic**

Center for Nanophase Materials Sciences Oak Ridge National Laboratory Oak Ridge, TN 37831, USA.

**Dr. Chaochao Dun, Dr. Jeffrey J. Urban**

The Molecular Foundry, Lawrence Berkeley National Laboratory, Berkeley, California 94720, United States of America.

**Dr. Samantha Husmann, Prof. Volker Presser**

INM – Leibniz Institute for New Materials, Campus D2 2, 66123, Saarbrücken, Germany.

**Prof. Volker Presser**

Department of Materials Science and Engineering, Saarland University, Campus D2 2, 66123, Saarbrücken, Germany.

saarene – Saarland Center for Energy Materials and Sustainability, Campus C4 2, 66123, Saarbrücken, Germany.

a) Present address: The State Key Laboratory of Fine Chemicals, School of Chemical Engineering, Dalian University of Technology, Dalian 116024, P. R. China.

b) Present address: Department of Chemical and Biomolecular Engineering, Vanderbilt University, Nashville, TN 37212, USA.


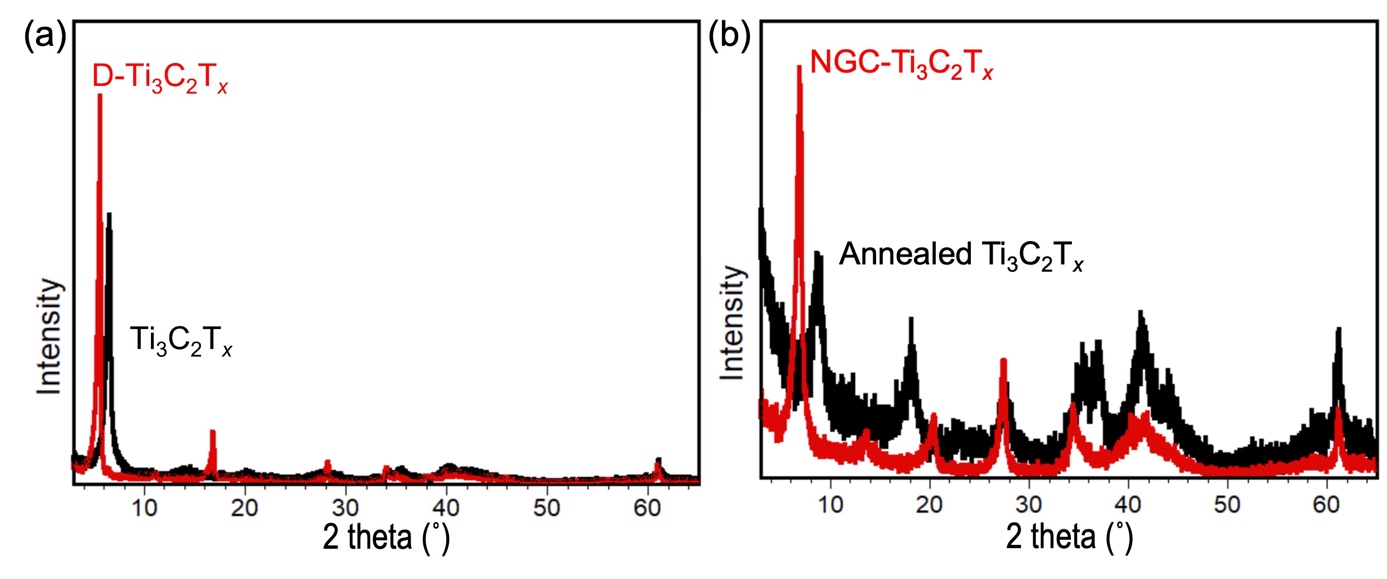


**Figure S1**. (a, b) X-ray diffractogramsbefore and after annealing treatment.


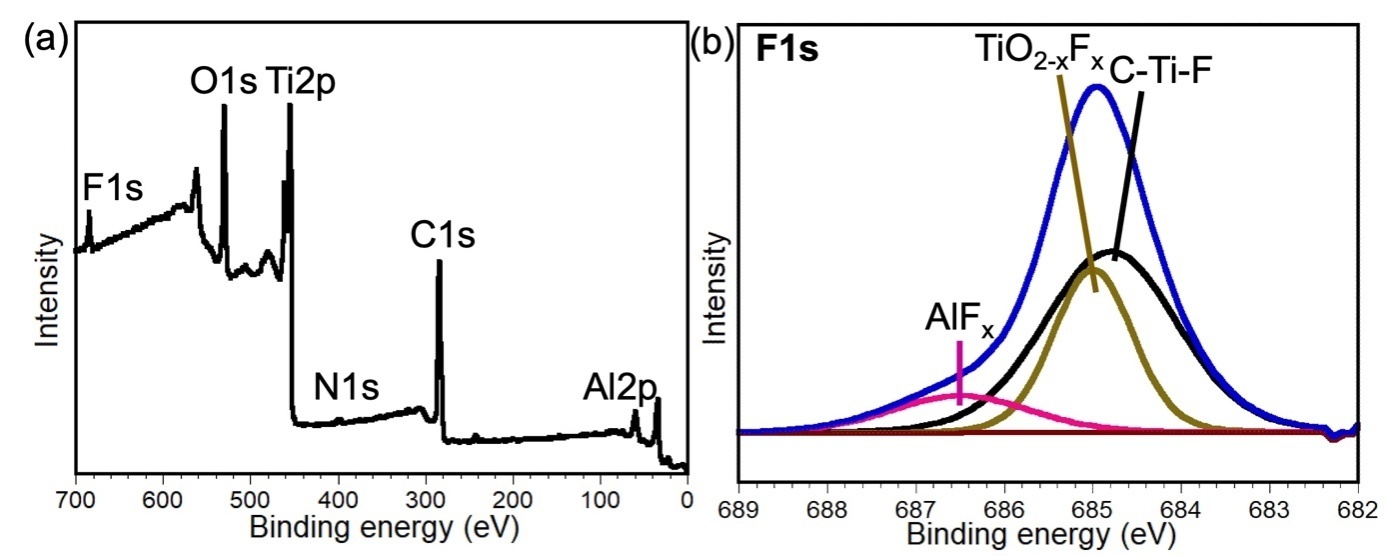


**Figure S2**. (a) XPS survey scan of NGC-Ti_3_C_2_T*_x_* and (b) High-resolution XPS spectra of F1s.


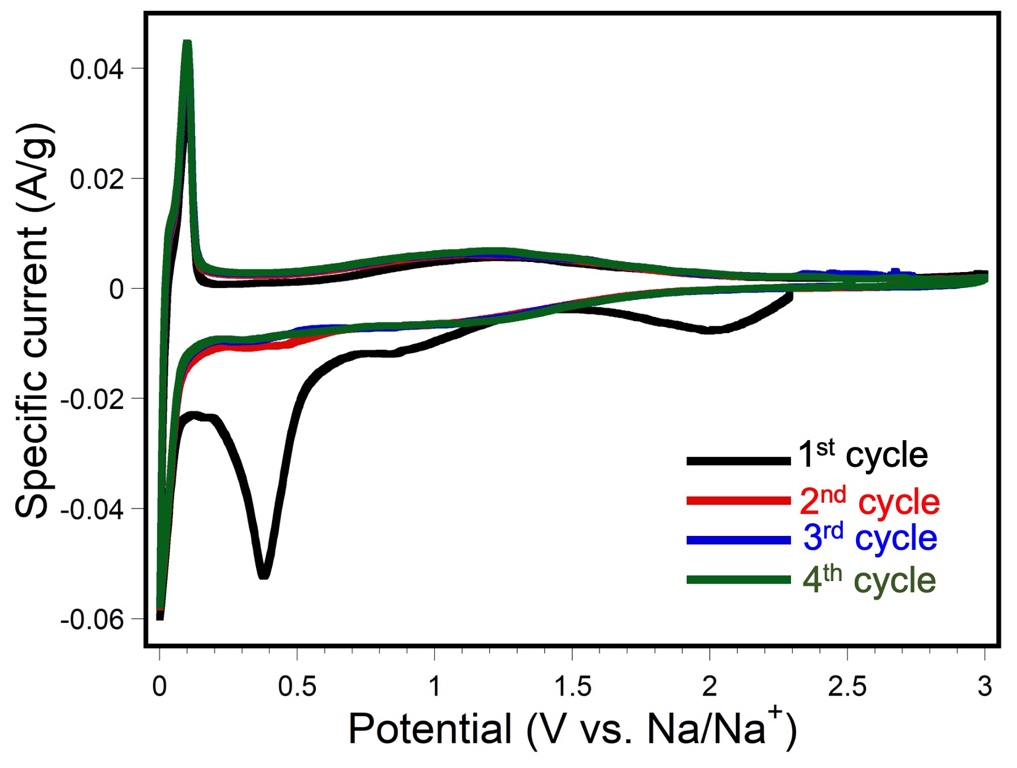


**Figure S3**. Cyclic voltammograms of Ti_3_C_2_T*_x_* for the four three cycles at a scan rate of 0.1 mV/s.


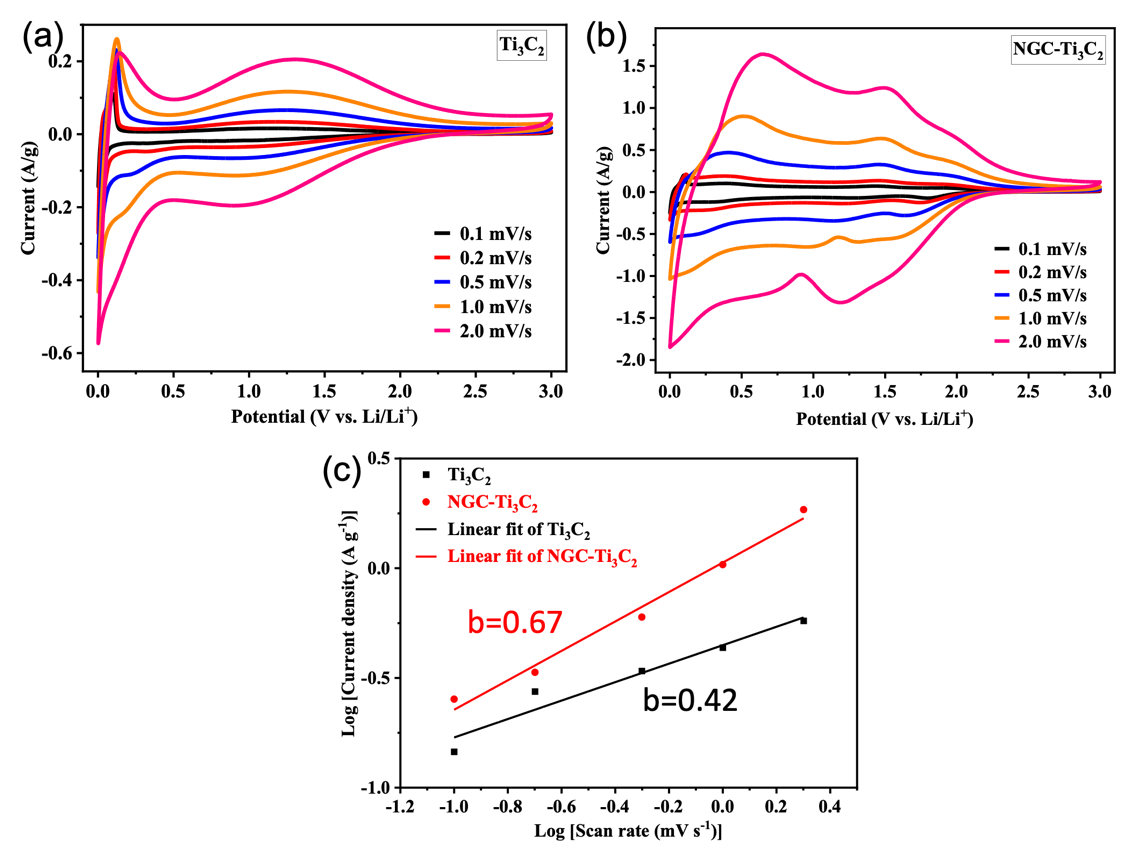


Figure S4. (a) CV curves of Ti_3_C_2_T*_x_* at various scan rates; (b) CV curves of NGC-Ti_3_C_2_T*_x_* at various scan rates; and (c) Determination of controlled steps, *b* values, through the logarithm of discharge peak current (*i*_p_) versus logarithm of scan rate (*ν*).


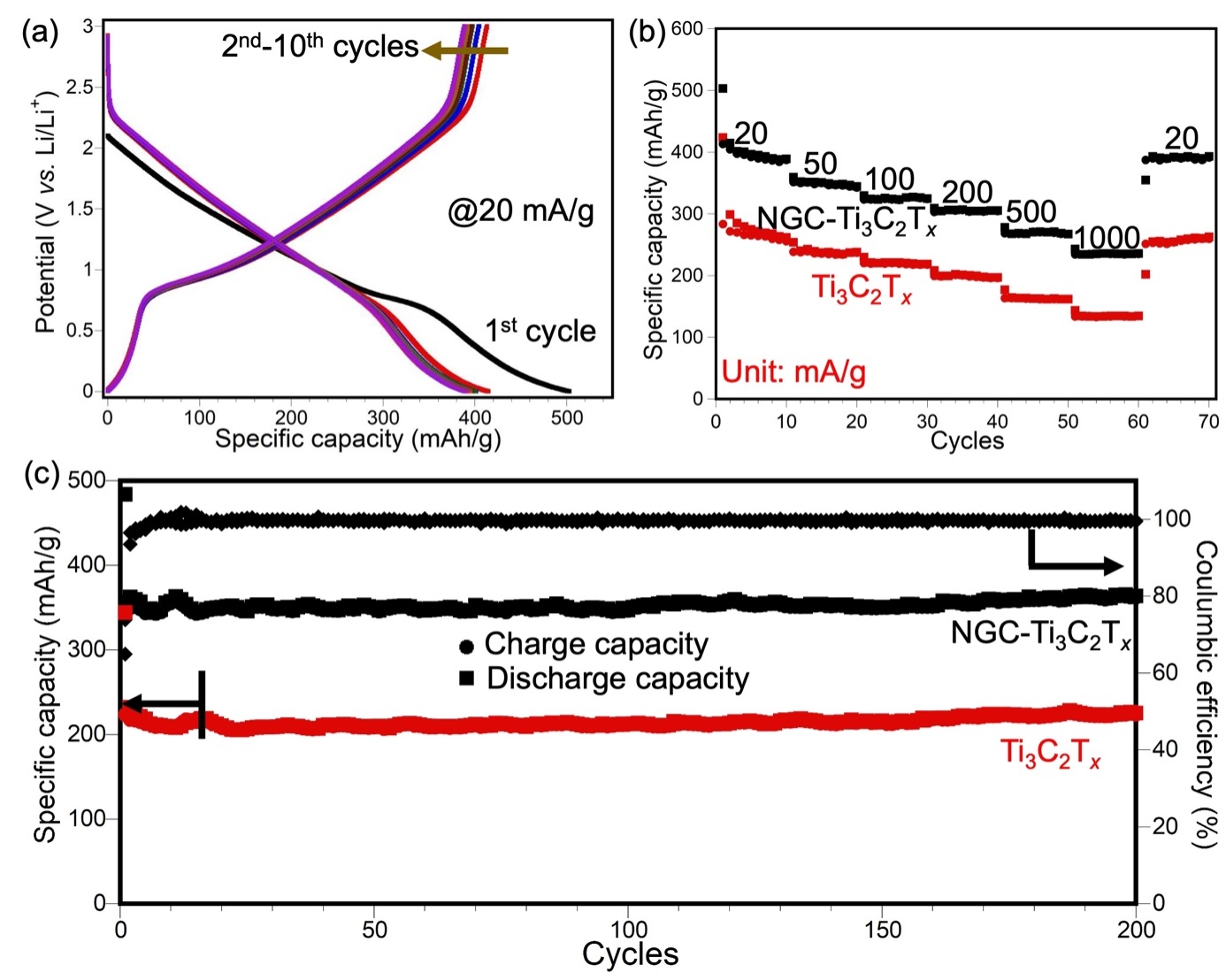


**Figure S5**. Electrochemical performance of NGC-Ti_3_C_2_T*_x_* as a Li-ion battery electrode material. (a) Galvanostatic charge/discharge testing of NGC-Ti_3_C_2_T*_x_* for the first ten cycles of at a specific current of 20 mA/g. (b) Rate capabilities of NGC-Ti_3_C_2_T*_x_* and Ti_3_C_2_T*_x_* as Li-ion battery electrodes. (c) Long-term cycling performance at a current density of 200 mA/g.


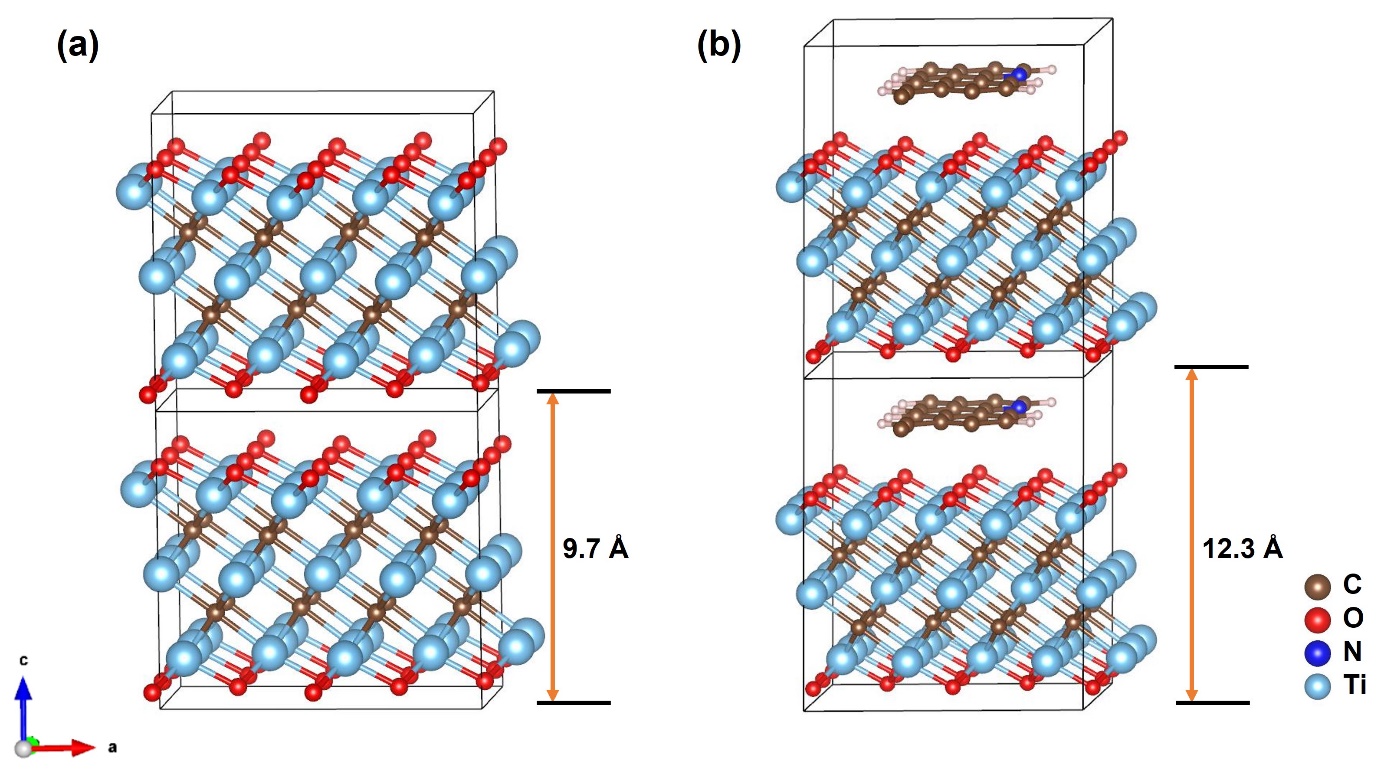


**Figure S6**. The optimized bulk structures of (a) Ti_3_C_2_O_2_ and (b) NGR/Ti_3_C_2_O_2_.


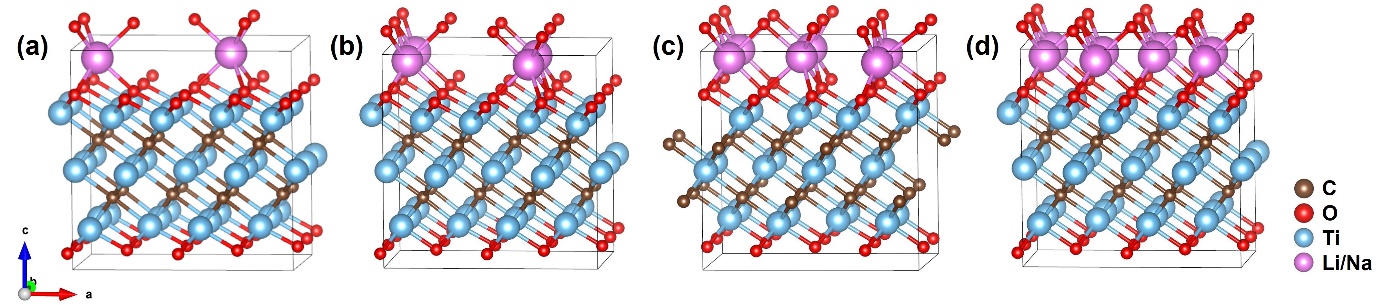


**Figure S7**. The optimized stable structures of Ti_3_C_2_O_2_ with different Li/Na-ion concentration: (a) Ti_3_C_2_O_2_-0.25Li/Na, (b) Ti_3_C_2_O_2_-0.50Li/Na, (c) NGC-Ti_3_C_2_O_2_-0.75Li/Na, and (d) Ti_3_C_2_O_2_-1.0 Li/Na.


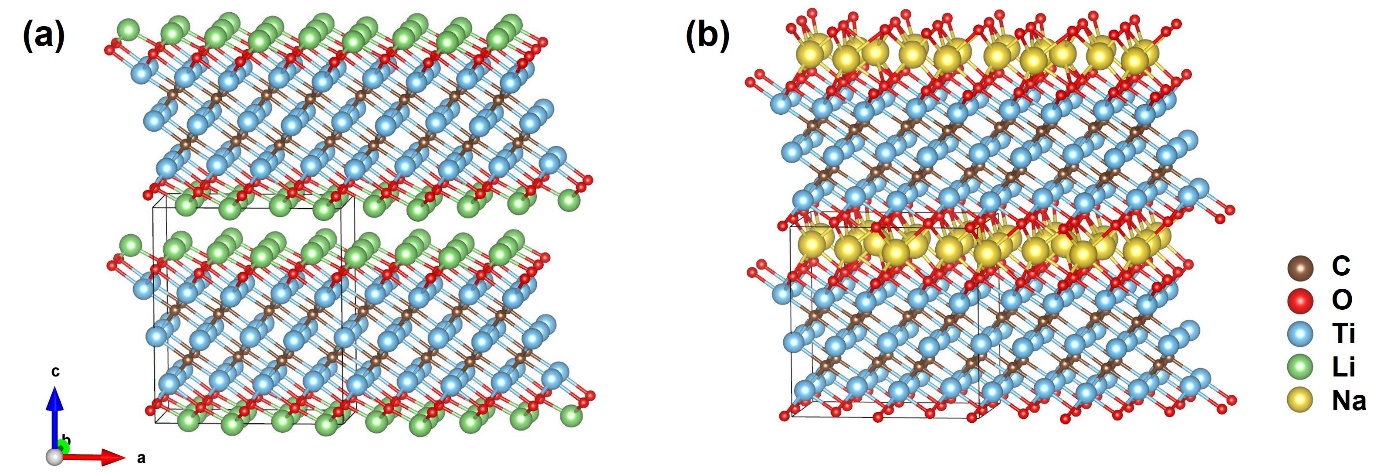


**Figure S8**. The optimized stable structures of Ti_3_C_2_O_2_ with maximum Li/Na-ion concentration: (a) Ti_3_C_2_O_2_-2.0Li, (b) Ti_3_C_2_O_2_-1.125Na.


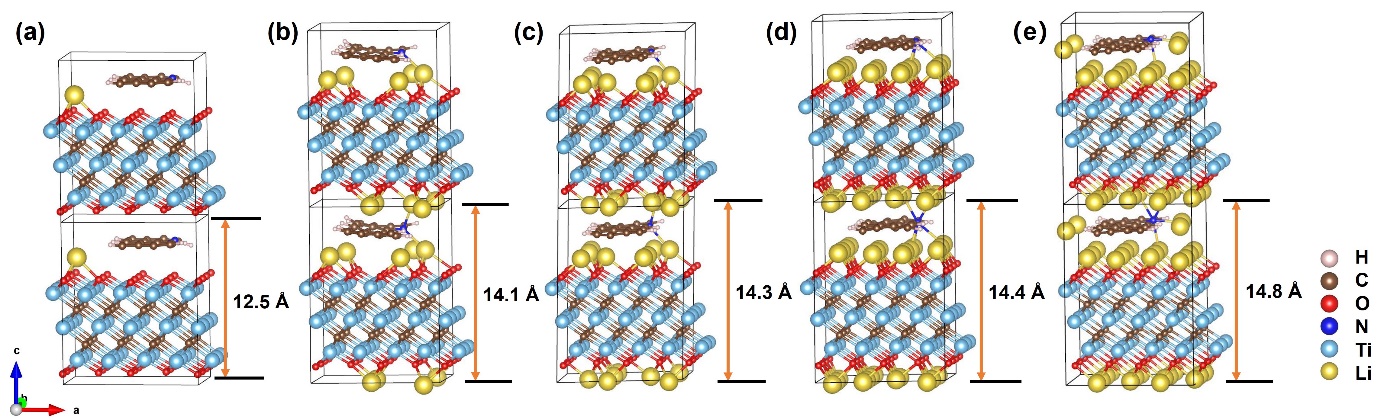


**Figure S9**. The optimized structures of NGC-Ti_3_C_2_O_2_ with different Na-ion concentration: (a) NGC-Ti_3_C_2_O_2_-0.083Na, (b) NGC-Ti_3_C_2_O_2_-0.667Na, (c) NGC-Ti_3_C_2_O_2_-1.0Na, (d) NGC-Ti_3_C_2_O_2_-2.0Na, and (e) NGC-Ti_3_C_2_O_2_-2.33Na.

**Table S1:** XPS fitting results of NGC-Ti_3_C_2_T*_x_*after sputtering. BE: binding energy. FWHM: full width at half maximum.

| **Region** | **BE (eV)** | **FWHM (eV)** | **Fraction** | **Assigned to** | **Ref.** |
| --- | --- | --- | --- | --- | --- |
| Ti 2p_3/2_ (Ti 2p_1/2_) | 454.68 (460.79) | 0.86 (1.32) | 0.18 | Ti-C | 1 |
|  | 455.22 (461.65) | 1.51 (1.44) | 0.26 | Ti (II) | 1 |
|  | 456.34 (462.65) | 1.64 (1.61) | 0.2 | Ti (III) | 1 |
|  | 457.5 (463.73) | 1.68 (2.02) | 0.16 | TiO_2_ | 1 |
|  | 458.68 (465.14) | 1.64 (2.29) | 0.11 | TiO_2-x_N_x_ |  |
|  | 459.8 (467.1) | 1.73 (2.95) | 0.09 | TiO_2-x_F_x_ | 1 |
| C 1s | 282.1 | 0.87 | 0.38 | C-Ti | 1 |
|  | 284.6 | 1.83 | 0.44 | C-C | 1 |
|  | 285.2 | 1.55 | 0.15 | C-O | 1 |
|  | 286.4 | 1.47 | 0.03 | C-N | 1 |
| N 1s | 396.4 | 1.39 | 0.43 | Ti-ON | 1 |
|  | 398.4 | 2.13 | 0.4 | Pyridinic N | 1 |
|  | 399.93 | 1.01 | 0.12 | Pyrrolic N | 2 |
|  | 401.0 | 1.05 | 0.06 | Graphite N | 2 |
| O 1s | 529.84 | 1.12 | 0.32 | TiO_2_ | 1 |
|  | 530.45 | 1.08 | 0.35 | TiO_2-x_F_x_ | 1 |
|  | 530.98 | 1.17 | 0.16 | C-Ti-O_x_/OR | 1 |
|  | 531.92 | 1.39 | 0.11 | C-Ti-(OH)_x_/OR | 1 |
|  | 533.1 | 1.66 | 0.06 | H_2_O_ad_/OR | 1 |
| F 1s | 684.8 | 1.78 | 0.57 | C-Ti-Fx (III) | 1 |
|  | 685.0 | 1.06 | 0.31 | TiO_2-x_Fx | 1 |
|  | 686.5 | 1.88 | 0.12 | AlFx/Al(OF)x | 1 |

Table S2 Comparison with other materials as LIBs/SIBs anodes

| Electrode materials | Applications | Current (A/g) | Specific capacity (mAh/g) | Ref. |
| --- | --- | --- | --- | --- |
| NGC-Ti_3_C_2_T_x_ | LIBs | 0.02 | 400 | This work |
| Ti_3_C_2_T_x_ | LIBs | 0.02 | 267 | This work |
| Carbon black/Ti_3_C_2_ | LIBs | 0.03 | 97 | ^[2]^ |
| Ti_3_C_2_-rGO | LIBs | 0.05 | 335.5 | ^[3]^ |
| TiO_2_/Ti_3_C_2_ | LIBs | 0.2 | 267 | ^[4]^ |
| MoS_2_/Ti_3_C_2_ | LIBs | 1 | 131.6 | ^[5]^ |
| Ti_3_C_2_/TiO_2_/rGO | LIBs | 1 | 176 | ^[6]^ |
| NGC-Ti_3_C_2_T_x_ | SIBs | 0.02 | 305 | This work |
| Ti_3_C_2_T_x_ | SIBs | 0.02 | 133 | This work |
| Na_0.23_TiO_2_/Ti_3_C_2_ | SIBs | 0.1 | 140 | ^[7]^ |
| Porous Ti_3_C_2_T_x_ monoliths | SIBs | 0.1 | 188 | ^[8]^ |
| Ti_3_C_2_T_x_/MoS_2_ | SIBs | 0.1 | 251 | ^[9]^ |
| Ti_3_C_2_T_x_ Nanoribbons | SIBs | 0.2 | 50 | ^[10]^ |
| Ti_3_C_2_T_x_ /TiO_2_ | SIBs | 0.96 | 116 | ^[11]^ |

**Supporting references**

[1] J. Halim, K. M. Cook, M. Naguib, P. Eklund, Y. Gogotsi, J. Rosen, M. W. Barsoum, Appl. Surf. Sci*.* 2016, 362, 406-417.

[2] S. J. Kim, M. Naguib, M. Zhao, C. Zhang, H.-T. Jung, M. W. Barsoum, Y. Gogotsi, Electrochim. Acta 2015, 163, 246.

[3] Z. Ma, X. Zhou, W. Deng, D. Lei, Z. Liu, ACS Appl. Mater. Interfaces 2018, 10, 3634.

[4] C. Yang, Y. Liu, X. Sun, Y. Zhang, L. Hou, Q. Zhang, C. Yuan, Electrochim. Acta 2018, 271, 165.

[5] D. Sun, M. Wang, Z. Li, G. Fan, L.-Z. Fan, A. Zhou, Electrochem. Commun. 2014, 47, 80.

[6] Z. Li, G. Chen, J. Deng, D. Li, T. Yan, Z. An, L. Shi, D. Zhang, ACS Sustain. Chem. Eng. 2019, 7, 15394.

[7] J. Huang, R. Meng, L. Zu, Z. Wang, N. Feng, Z. Yang, Y. Yu, J. Yang, Nano Energy 2018, 46, 20.

[8] J. Zhao, Q. Li, T. Shang, F. Wang, J. Zhang, C. Geng, Z. Wu, Y. Deng, W. Zhang, Y. Tao, Q.-H. Yang, Nano Energy 2021, 86.

[9] Y. Wu, P. Nie, J. Jiang, B. Ding, H. Dou, X. Zhang, ChemElectroChem 2017, 4, 1560.

[10] P. Lian, Y. Dong, Z.-S. Wu, S. Zheng, X. Wang, W. Sen, C. Sun, J. Qin, X. Shi, X. Bao, Nano Energy 2017, 40, 1.

[11] X. Guo, J. Zhang, J. Song, W. Wu, H. Liu, G. Wang, Energy Storage Mater. 2018, 14, 306.
